# Multicriteria site suitability for solar-powered green hydrogen production plants along the Northwestern coast of Egypt

**DOI:** 10.1038/s41598-026-44081-8

**Published:** 2026-04-14

**Authors:** Abdel-hameed M. El-Aassar, Kamilia H. Hagagg, Rasha A. Hussien

**Affiliations:** 1 Desert Research Center, Cairo, Egypt; 2 Nuclear and Radiological Safety Research Center, Egyptian Atomic Energy Authority, Cairo, Egypt

**Keywords:** Green-hydrogen, Siting suitability, Environmental isotopes, Fuzzy-analytical hierarchy process, Marsa Matruh, Egypt, Environmental sciences, Hydrology, Solid Earth sciences

## Abstract

An integrated geospatial, hydrogeochemical framework for identifying environmentally suitable locations for solar-powered green hydrogen production along Egypt’s northwestern Mediterranean coast is applied. The methodology combines environmental stable isotope of groundwater (δ^1^⁸O and δ^2^H) for quaternary, upper miocene, and lower miocene aquifers with multi-criteria spatial analysis in geographic information system (GIS). Isotopic signatures are used to evaluate groundwater recharge sources, salinization processes, and the extent of seawater intrusion providing a quantitative basis for assessing aquifers vulnerability. Eight spatial criteria, elevation, slope, aspect, land use/land cover, proximity to roads, proximity to the shoreline, hydraulic head, and seawater intrusion index, are weighted using fuzzy-analytic hierarchy process (AHP) and integrated to generate site suitability map for hydrogen infrastructure development. The results classify the study area into five suitability zones, with central and northeastern sectors emerging as the most favorable due to high solar potential, lower groundwater vulnerability, and adequate accessibility. Environmentally constrained zones are primarily associated with low hydraulic heads and pronounced seawater intrusion near the coast. At the regional scale, the framework supports informed decision-making for sustainable hydrogen deployment while minimizing impacts on fragile coastal aquifers. More broadly, the study demonstrates the value of coupling isotope hydrology, hydrochemistry and environmental parameters with GIS-based decision analysis, offering a transferable approach for siting renewable hydrogen projects in arid and semi-arid coastal regions worldwide.

## Introduction

Across recent global energy and climate research, green hydrogen is consistently identified as a strategic pillar of the low-carbon transition. Unlike gray or blue hydrogen, green hydrogen is produced via water electrolysis powered by renewable energy (solar, wind, hydro), resulting in near-zero lifecycle CO₂ emissions. Scholars emphasize its unique role in decarbonizing hard-to-abate sectors, integrating variable renewables, strengthening energy security, and enabling new global clean-energy trade routes^1–7^. While cost and infrastructure barriers remain^8^, the literature agrees that large-scale green hydrogen deployment is essential to achieving net-zero targets by mid-century. In accordance with international climate agreements like the Paris Agreement. The global energy environment must be fundamentally restructured in order to meet the 1.5 °C objective. In this regard, green hydrogen, produced has become a crucial route for deep decarbonization, particularly in industries like heavy industry, aviation, and maritime transportation where emissions are hard to reduce^9^. It is positioned as a key component of future low-carbon economies due to its adaptability and scalability^10^. However, the expansion of green hydrogen infrastructure at scale requires careful spatial planning to reconcile environmental sustainability with economic viability.

Strategic site selection is critical to optimize resource use, minimize ecological disruption, and align with national and regional development priorities^11^. Robust, spatially informed methodologies are essential for identifying locations that support long-term climate and sustainability goals while accounting for environmental constraints. Recent academic literature converges on the view that Egypt is one of the most strategically positioned countries for green hydrogen production in the Middle East and North Africa (MENA). Studies highlight Egypt’s exceptional solar and wind resources, strategic geographical location, expanding energy infrastructure, and strong governmental commitment (Vision 2030 and post-COP27 initiatives). Green hydrogen is widely seen as a catalyst for Egypt’s energy transition, industrial decarbonization, export-led growth, and regional leadership in clean energy. Egypt’s national hydrogen strategy identifies solar-powered hydrogen production as a central component of its energy transition and export vision^12^. The northwestern coast of Egypt offers particularly favourable conditions, including high solar irradiance exceeding 2600 kWh/m^2^ annually^13^, vast tracts of undeveloped land, and proximity to Mediterranean trade routes. These attributes support the potential for large-scale hydrogen generation and export, with access to existing transmission infrastructure and major ports facilitating distribution to European and Asian markets. Despite these advantages, the region faces notable environmental challenges. High initial capital costs for electrolyzers, water resource management (desalination integration), infrastructure adaptation for hydrogen transport and storage, fragile coastal ecosystems, vulnerable aquifers, and risks of land degradation present significant constraints to development^14^. These factors underscore the need for rigorous site evaluation that integrates both technical and ecological considerations. Some previous studies apply multiple datasets to identify and categorize suitable areas for green hydrogen production^15,16^, Eddine et al., 2023,^17^. The integration of satellite image analysis with GIS mapping enhances the strategic approach and improves understanding of results^18^. Statistical methodologies like the Analytic hierarchy process (AHP) increase the accuracy of results^19^, while combining analytic hierarchy process results with geophysical data substantially boosts reliability of the results^20,21^. Leveraging remote sensing, geophysical methods and AHP model to determine optimal locations for green hydrogen production. AHP and Fuzzy AHP together provide a robust framework for site suitability analysis, balancing the clarity of AHP with the adaptability of fuzzy logic. AHP’s effectiveness has been demonstrated in several studies, while Fuzzy AHP has been widely used to enhance decision-making where uncertainty is present, making both methods suitable for this research^22^.

Conventional multicriteria assessments typically rely on surface-level GIS analysis, with limited attention to hydrogeological processes such as groundwater salinization and seawater intrusion, factors that require detailed isotopic and geochemical investigation. Moreover, while an organized method is provided by Multi-Criteria Decision Analysis (MCDA) to integrating diverse criteria, its application to hydrogen planning in Egypt remains underexplored. Few studies incorporate Environmental Impact Assessment (EIA) or stakeholder input in evaluating coastal hydrogen infrastructure^23–25^. A previous study including the study area was applied for Techno-economic assessment of using hybrid renewable energy system of wind turbine and photovoltaic (PV) panels for hydrogen production and storage at different climate conditions of five different Egyptian cities^26^. Concluding that the produced annual solar radiation and wind energy are equal to 248.6 MWh in Marsa Matruh and the amount of electricity produced from the hybrid system ranges from 69,836.5 kWh in El Arish to 118,115 kWh in Marsa Matruh., so the annual hydrogen production reaches 1,972 kg for Maras Matruh. This study suggests a spatially explicit approach for locating the best locations for solar-powered green hydrogen generation along Egypt’s northwest coast in order to close these gaps. The methodology integrates Fuzzy- analytic hierarchy process (FAHP) within a GIS environment to combine technical, environmental, and economic factors into a comprehensive decision-support tool. Stable isotope analysis (δ^1^⁸O and δ^2^H) is employed to assess groundwater recharge mechanisms, salinization trends, and seawater intrusion, providing critical insights into aquifer vulnerability. Eight spatial criteria: slope, aspect, elevation, land use/land cover, proximity to roads and shoreline, hydraulic head, and seawater intrusion index, are weighted and synthesized to generate a composite suitability map. The results identify zones that combine high solar potential and infrastructure access with minimal environmental stress. Beyond its regional relevance, the proposed framework offers a transferable model for sustainable hydrogen development in other arid and semi-arid coastal regions, contributing to global efforts in climate adaptation and energy transition.

## Site description

**Fig. 1:**
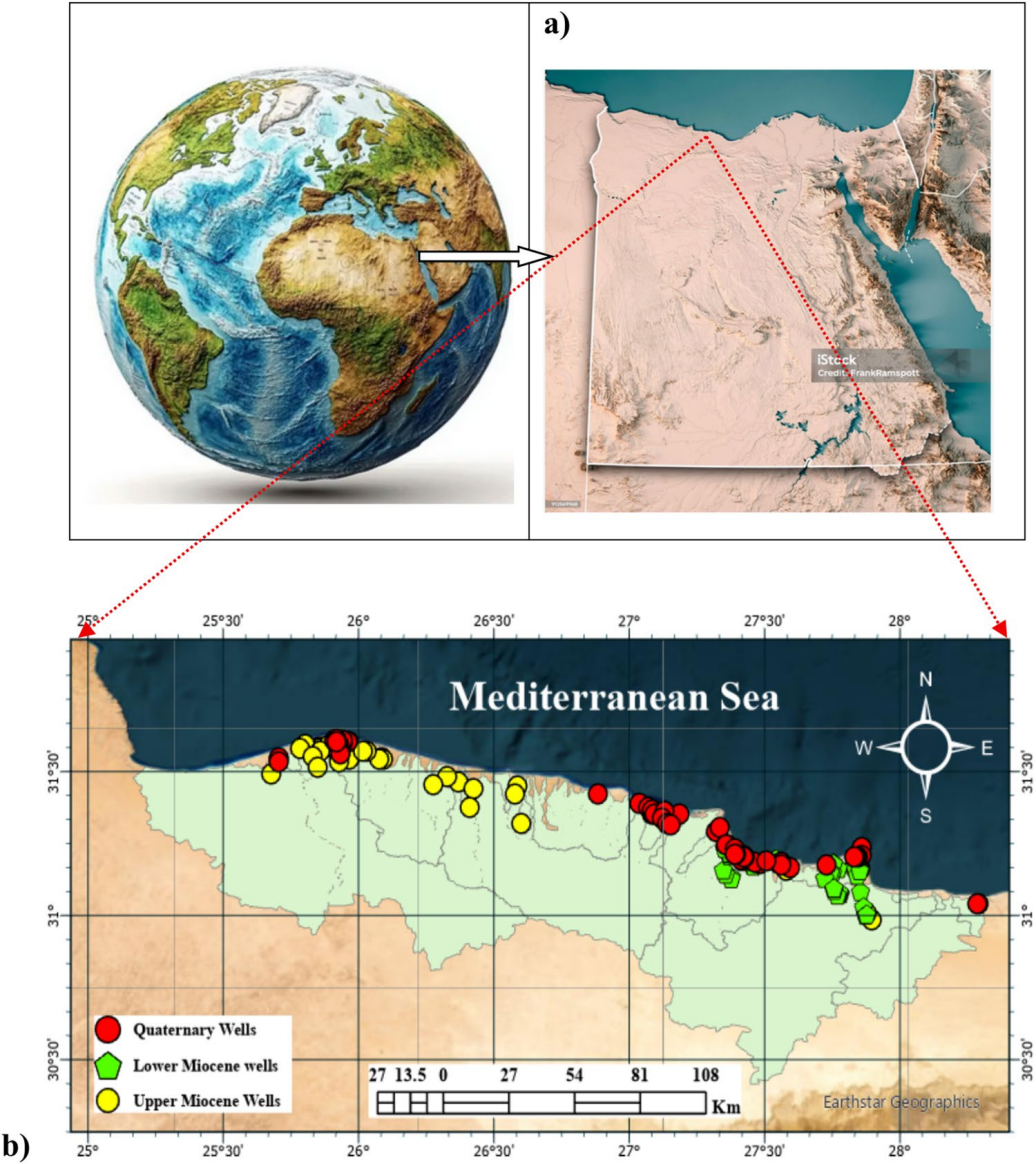
(**a**) Map of the study area; (**b**) Location of water sampling wells (the map was created using QGIS (QGIS Development Team, 2025, Version: 3.34.6-Prizern. Available at: https://qgis.org).

Marsa Matrouh Governorate is located at the northern west of Egypt as shown in Fig. 1a,b between longitude 30° 14` 58.5‶ and 31° 57` 28.08‶E and latitude 24° 56` 12.2‶ and 28° 24` 15.3‶N; its coastline is 500 km long with coastal area covers about 15000 km^2^. Within this area there is El Omayad Protectorate with an area of 700 km^2^, in addition to several other areas which have been surveyed and are being nominated as protected areas, including about 2500 km^2^ area between Matrouh and Salum. The area has some of the highest annual rainfall in the country. The relatively high precipitation (up to 170 mm) gives rise to a rich belt of vegetation along the coast, which becomes gradually sparser as one travel south turning into the hyper-arid terrain characteristic of the Western Desert. There are four agro-ecological zones from north to south—firstly, an agricultural belt in the coastal strip, where rainfall is highest, populations are settled and the area has good soils for irrigated fruit cultivation. Secondly, a mixed production strip ranging 5–15 km inland where the population is settled with a mixed sheep/goat herdingcum- barley farming system. Thirdly, a rangeland strip where the lack of water is limiting the livestock and agriculture activates and fourthly, a desert ecosystem with true nomads prevails. The coastal area under consideration is shared by two governorates, namely: Alexandria and Matrouh. However, the area of focus is Marsa Matrouh, which is the capital of Matrouh Governorate. It has a population of 50,000 capita and the total population of the area is estimated at 227,840 capita of which 85% are Bedouin,^27^.

### Geomorphological and geological aspects

**Fig. 2:**
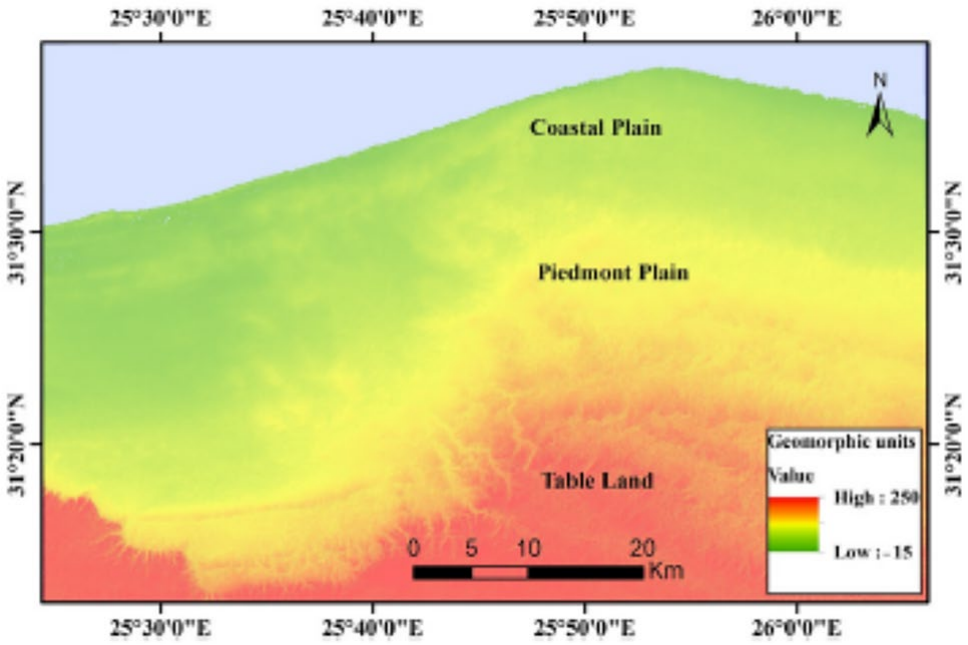
Geomorphological units.

Geomorphologically, the landforms within the study area reflect the influences of endo-genetic factors (e.g., faulting, folding, lithologic features, etc.) and exo-genetic factors (e.g., climatic conditions, weathering, deposition, erosion, etc.)^28,29^. These factors result in the emergence of various landforms, such as tablelands, ridges, depressions, and dunes as well as drainage lines, all of which are affected by the distribution of surface runoff in addition to the accumulation and storage of groundwater. Three main geomorphologic units were identified: the southern tableland, the piedmont plain, and the coastal plain^30^ (Fig. 2). The southern tableland extends southwards to the Qattara Depression, with a maximum elevation of 250 m above sea level. The northern bounding slopes of this tableland are usually dissected by the drainage lines that discharge to the coastal plain. The piedmont plain, with low land and hills, is a transitional zone between the tableland to the south and the coastal plain to the north. Its elevation ranges from 30 to 90 m above sea level, while it varies between 2 and 25 km wide. The inland depressions can be seen within this plain in between the ridges^31^. The coastal plain occupies a narrow strip of land that extends parallel to the Mediterranean Sea, with elevations ranging from 0 to 50 m above sea level. The existence of alternating low-lying ridges separated by narrow depressions along the coast reflects the influence of lithologic and structural conditions as well as the fluctuation of sea level^32^.

**Fig. 3:**
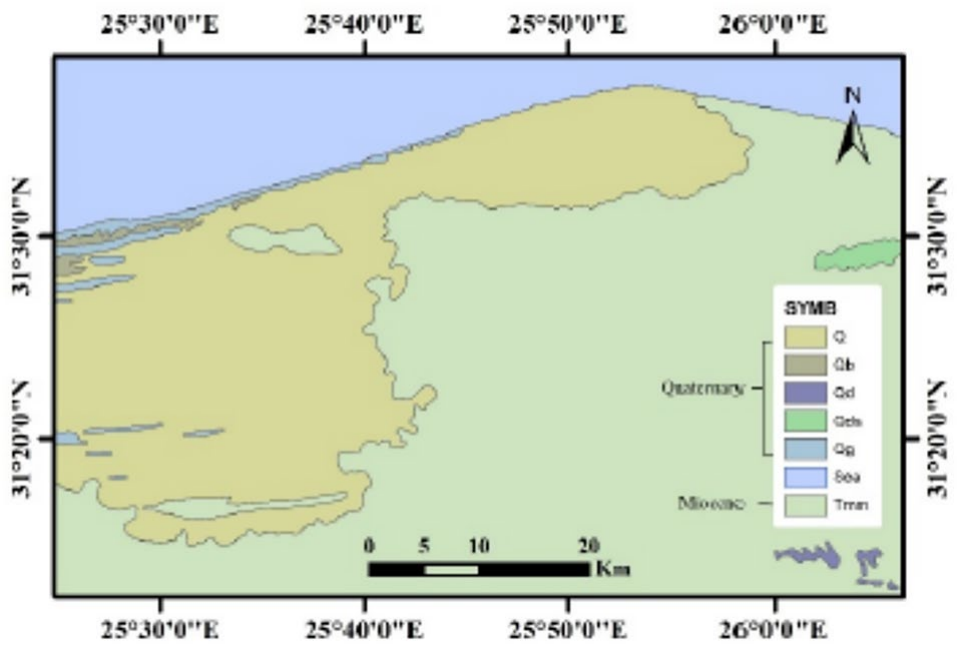
Surface geology map.

Geologically, the study area, which is a part of the northwestern Mediterranean coast, is mainly underlain by sedimentary rocks ranging in age from Tertiary to Quaternary^32^. The Miocene deposits are represented by Moghra and Marmarica Formations. The Moghra Formation is composed of argillaceous limestone intercalated with sand and shale related to fluviatile to fluviomarine deposits, while the overlying shallow marine rocks belong to the Marmarica Formation. It is formed of fracture white limestone and a lower grey calcarenite interbedded with clay lenses. The overlaying Quaternary deposits are exposed in the study area. They are formed by a thin cover of drift sands and loamy deposits covering mainly low-lying areas and the floors of narrow valleys dissecting the tableland (Fig. 3). The study area’s climatic conditions are typically arid, with a long, hot, dry summer, a mild winter with little rainfall, high evaporation, and moderate to high relative humidity, with a mean annual rainfall of 155 mm^33^.

Hydrologically, the Quaternary and Miocene aquifers are considered as the two main water-bearing areas under consideration. The Quaternary carbonate aquifer comprises wadi fill and oolitic limestone deposits, with a thickness ranging from 10 m to about 40 m^34,35^. It is mainly recharged the precipitation over its outcropping rocks. The Quaternary aquifer is directly connected to the Mediterranean Sea, which has a significant impact on groundwater salinity. Groundwater, in this aquifer, exists under unconfined conditions The Miocene limestone aquifer of middle Miocene age (Marmarica Formation) is the main water-bearing formation in the study area. It is mainly composed of successions of chalky, marly, argillaceous, and dolomitic limestones interbedded with clay lenses. The groundwater in this aquifer exists under two conditions: perched groundwater, where the water level is above mean sea level, and main water table, where the water is free or semi-confined. The perched water table is recharged indirectly by the overlying fractured rocks, while the fractured free to semi-confined aquifer is indirectly recharged by natural groundwater movement from south to north^35^. The degree of recharge depends on the nature of the fault from which this aquifer derives its water. Despite the fact that there is a general relationship between water level and salinity because salinity frequently rises with the direction of water flow, shows that the relationship between the two variables is not clearly defined in each of the Quaternary and Miocene aquifers. This characteristic could mean that other hydrological factors, rather than the absolute water level, have a significant impact on the salinity of the water, as is the case in some localities within the study area, which showed perched aquifer conditions. In general, the study area shows a general direction of groundwater flow from southeast to northwest.

## Materials and methods

### Multicriteria site suitability for a solar-powered green hydrogen production

#### Criteria selection and justification

The identification and selection of criteria constitute the foundation of any multi-criteria site suitability analysis. For solar-powered green hydrogen production, the criteria must capture the interconnected technical, economic and environmental dimensions that determine project feasibility. Following established frameworks in renewable energy siting literature, the criteria were organised into three categories, technical, economic and environmental, as this structure enables systematic evaluation of the diverse factors influencing hydrogen production facilities^36,37^.

##### Technical criteria

Direct normal irradiation (DNI) and average temperature represent the primary technical determinants of solar-based energy generation, governing the electricity output available to power electrolysis^38^. However, preliminary characterisation of the study area revealed negligible spatial variation in these climatic parameters across the region. This homogeneity, attributable to the limited geographical extent and consistent top climatic conditions, is a recognised scenario in regional-scale assessments wherein certain criteria cease to function as discriminating variables^39^. Under such conditions, technical criteria serve to establish baseline feasibility rather than differentiate between candidate locations. The entire study area satisfies the minimum technical requirements for solar-powered hydrogen production, rendering these factors unsuitable for ranking purposes. This approach follows established practice in multi-criteria decision analysis, where spatially invariant criteria may be treated as constraints or assigned minimal weights to prevent dilution of genuinely discriminating factors^40^. Consequently, the analytical weight shifts to economic and environmental criteria, which exhibit meaningful spatial heterogeneity and therefore determine relative site suitability within the region.

##### Economic criteria

Elevation affects both construction logistics and equipment performance. Higher elevations may confer efficiency benefits through reduced ambient temperatures but simultaneously increase transport costs and logistical complexity^39^. Consistent with previous studies, elevation receives lower weighting than slope and aspect, reflecting its secondary role in site suitability determinations^41^.

Slope imposes fundamental constraints on construction feasibility and capital expenditure. Steep terrain necessitates increased earthworks, complicates panel mounting systems and elevates installation costs. Recent suitability assessments for photovoltaic installations in Iraq identified slope as a high-priority criterion, with preference consistently assigned to flatter terrain to minimise civil engineering requirements^39^. For hydrogen production facilities, which accommodate not only solar arrays but also processing equipment and storage infrastructure, moderate slopes are prerequisite for economic development.

Aspect influences the solar radiation incident on sloping terrain, particularly in topographically varied landscapes. In the northern hemisphere, south-facing orientations receive enhanced insolation, improving energy yield^41^. While aspect is less critical on level ground, its inclusion ensures appropriate evaluation of areas where topography modifies the effective solar resource.

Proximity to roads is critical for economic viability. Hydrogen production facilities require access during construction for equipment delivery and throughout operation for maintenance and product transport. The emergence of dedicated hydrogen trucking corridors, exemplified by the 1,150 km route developed in China, underscores the importance of road infrastructure for hydrogen logistics^42^. Accessibility directly influences both capital and operational expenditures.

Proximity to shoreline addresses two strategic considerations. Coastal locations facilitate hydrogen export via maritime transport, as demonstrated by the Maritime Hydrogen Highway project developed by port authorities in the United Kingdom^43^. Additionally, coastlines provide access to seawater for electrolysis feedwater, though desalination requirements must be incorporated into cost calculations. This criterion therefore captures advantages for facilities oriented toward export markets.

##### Environmental criteria

Land use and land cover constitute fundamental environmental constraints governing the compatibility of hydrogen production with existing landscape functions. Photovoltaic siting studies in Türkiye identified land use as the most influential criterion, with agricultural lands and protected areas appropriately excluded or assigned low suitability^41^. The preservation of food-producing land and natural habitats aligns with the sustainability principles underlying green hydrogen production.

Seawater intrusion and hydraulic head are groundwater-related criteria that reflect the critical importance of water availability for electrolysis. Hydrogen production via water electrolysis requires approximately 15–170 L of water per kilogram of hydrogen, depending on feedwater quality and cooling system configuration^44^. In coastal aquifers, seawater intrusion compromises freshwater availability, while hydraulic head indicates groundwater accessibility and sustainable yield. These criteria ensure that site selection considers long-term water resource availability, a factor frequently overlooked in renewable energy siting studies.

These criteria focus on economic and environmental criteria, justified by the spatial homogeneity of technical factors within the study area, enables identification of sites that are not only technically feasible but also economically viable and environmentally responsible.

### Analytic hierarchy process (AHP) for criteria weighting

**Fig. 4:**
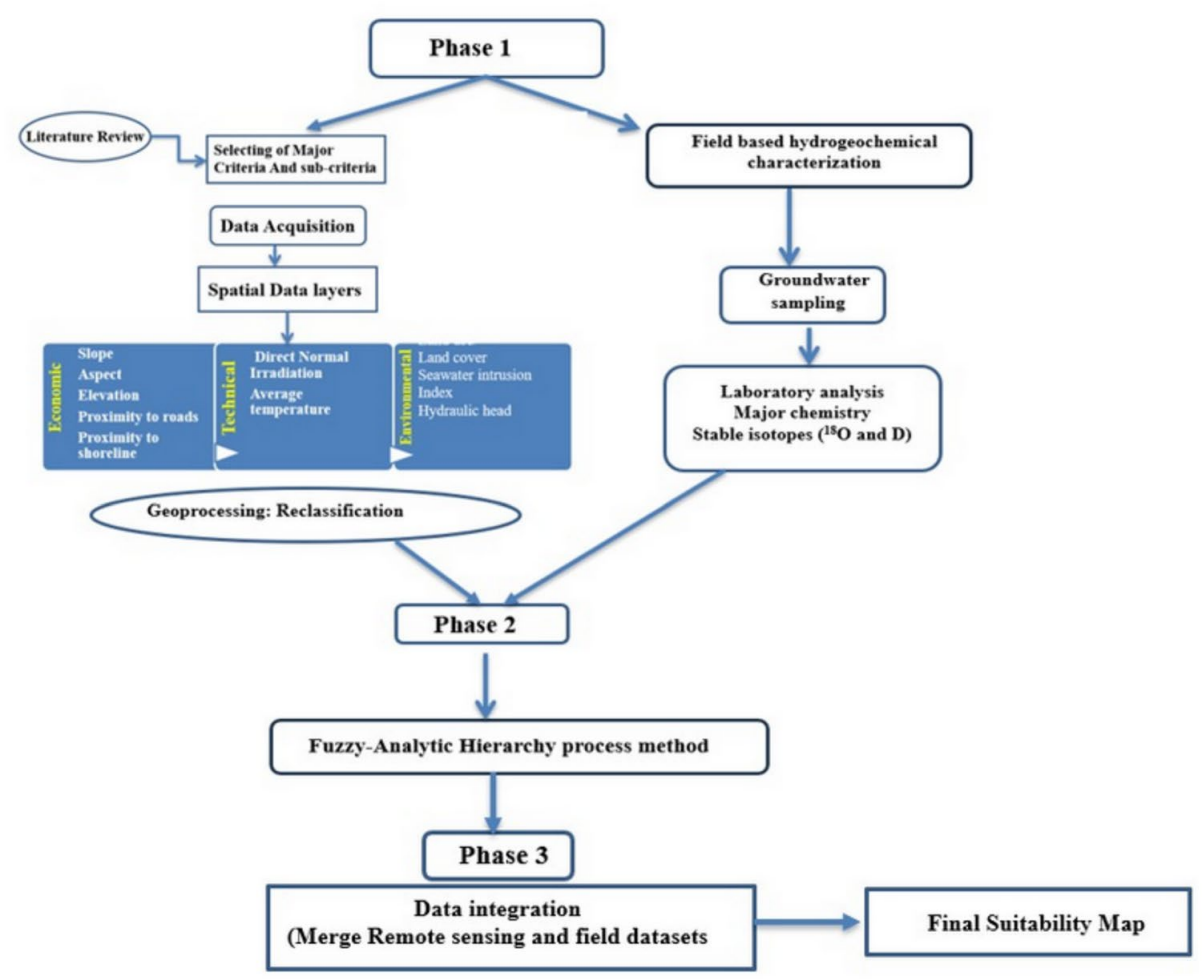
Methodological approach for solar-hydrogen site selection.

The analytic hierarchy process (AHP), developed by Thomas Saaty in 1977, is a structured multi-criteria decision-making framework that decomposes complex problems into a hierarchy of objectives, criteria, sub-criteria, and alternatives. The steps involved in the AHP process are illustrated Fig. 4. Was employed to identify optimal sites for solar power plants dedicated to green hydrogen production worldwide^15–17,45,46^. In this study, based on the eight solar energy experts from academia and industry were invited to complete a pairwise comparison matrix, evaluating criteria and sub-criteria. They assigned values from 1 to 9, indicating the relative importance of each parameter in comparison to others. Eight primary criteria, Slope, Aspect, Elevation, Distance to Roads, LULC, Distance to shoreline, Hydraulic Head, Seawater Intrusion Index in addition to solar radiation were selected based on literature review and stakeholder consultations. Pairwise comparisons were conducted under the analytic hierarchy process to calculate normalized eigenvector weights, ensuring all consistency ratios (CR) remained below 0.10 as illustrated in Table 1.

**Table 1:** Pairwise comparison matrix of site selection criteria.

Criteria	Shoreline	Intrusion	Head	Roads	LULC	Slope	Aspect	Elevation
Distance to shoreline	1	1	3	3	5	7	7	7
Seawater intrusion index	1	1	3	3	5	7	7	7
Hydraulic head	1/3	1/3	1	1	3	5	5	5
Distance to roads	1/3	1/3	1	1	3	5	5	5
Land use/land cover (LULC)	1/5	1/5	1/3	1/3	1	3	3	3
Slope	1/7	1/7	1/5	1/5	1/3	1	1	1
Aspect	1/7	1/7	1/5	1/5	1/3	1	1	1
Elevation	1/7	1/7	1/5	1/5	1/3	1	1	1

The consistency ratio (CR) was calculated using the conventional AHP approach to guarantee the validity of expert assessments in the pairwise comparison matrix. First, the principal *eigenvalue (λ*max) was derived from the matrix, yielding a value of approximately 8.42 for the eight selected criteria. The Consistency Index (CI) was *then calculated to be 0.06*. To assess the acceptability of this consistency level, the CI was compared against the Random Index (RI), which is 1.41 for eight parameters. The resulting Consistency Ratio is ≈ 0.08. Since the CR value is below the threshold of 0.10, the judgments are considered consistent and suitable for deriving reliable criterion weights. This confirms the internal coherence of the expert evaluations and validates the use of the resulting weights in subsequent spatial analysis. The resulting priority levels and normalized weights reflect a structured evaluation of technical feasibility, groundwater protection, and coastal accessibility*.* As shown in Table 2, Distance to Shoreline *and* Seawater Intrusion Index were assigned the highest priority (0.22 each), underscoring their dual role in ensuring seawater availability while minimizing aquifer vulnerability. Hydraulic Head and Distance to Roads followed with substantial weights (0.15), indicating their relevance to groundwater sustainability and logistical access. Land Use/Land Cover (LULC) was moderately weighted (0.10), reflecting land availability and potential environmental conflict. Topographic parameters, Slope*,* Aspect*, and* Elevation, received lower weights (0.053 each), suggesting limited influence under the study’s coastal context. These weights were integrated into a GIS-based weighted overlay to generate the composite suitability index.

**Table 2:** Relative priority and normalized weight of the selected parameter.

Criterion	Relative priority	Normalized weight
Distance to shoreline	Very high	0.22
Seawater intrusion index	Very high	0.22
Hydraulic head	High	0.15
Distance to roads	High	0.15
LULC	Moderate	0.10
Slope	Low	0.053
Aspect	Low	0.053
Elevation	Low	0.053

While AHP relies on precise numerical judgments, many real-world evaluations involve uncertainty and imprecision. Fuzzy Set Theory provides a means to represent vagueness by assigning degrees of membership to values rather than binary classifications. This allows subjective judgments to be expressed using linguistic terms (e.g., “high importance,” “moderate importance”) and modeled through fuzzy numbers. Fuzzy AHP (FAHP) combines the hierarchical structure of AHP with the flexibility of fuzzy logic, thereby addressing uncertainty in expert evaluations. Instead of crisp numerical inputs, FAHP employs fuzzy numbers to capture the range of possible preferences, producing more nuanced and robust weightings of criteria. This integration is particularly relevant in environmental and energy planning, where expert opinions often involve ambiguity.

### Integration of AHP and fuzzy membership in GIS-based MCDA

In this study, the analytic hierarchy process (AHP) is employed to calculate the relative weights of the selected criteria. AHP provides a structured framework for expert-based pairwise comparisons, ensuring consistency in judgments and producing normalized eigenvector weights^47^. These weights represent the relative importance of slope, aspect, elevation, land cover, distance to roads, distance to shoreline, hydraulic head, seawater intrusion index, and solar radiation in determining site suitability. While AHP determines the weights, the fuzzy membership approach is applied to reclassify each spatial dataset into a continuous suitability scale ranging from 0 (least suitable) to 1 (most suitable). Fuzzy membership functions allow gradual transitions between categories, capturing the inherent uncertainty and variability in environmental parameters. The following Table 3 represent the fuzzy membership function and their suitability logic.

**Table 3:** Thematic layers and fuzzy membership functions.

Thematic layer	Fuzzy membership function	Suitability logic
Slope	Linear decreasing	Gentle slopes (0–5°) = high suitability (1); steep slopes (> 20°) = low suitability (0)
Aspect	Gaussian centered at 180° (south)	South-facing slopes = maximum suitability (1); suitability decreases symmetrically toward north (0°/360°)
Elevation	Sigmoidal	Moderate elevations (50–200 m) = optimal; very low (< 20 m, flood risk) or very high (> 400 m, construction difficulty) = low suitability
Distance to roads	Linear increasing	Areas closer to roads (< 2 km) = high suitability; remote areas (> 10 km) = low suitability
Land Cover (Sentinel-2)	Fuzzy categorical	Barren/unused land = high suitability (1); agricultural land = moderate (0.5); urban/forested = low (0)
Distance to shoreline (Sentinel-2)	Sigmoidal	Moderate distance (5–15 km) = optimal (balance between access and coastal vulnerability); very close (< 2 km) or very far (> 30 km) = low suitability
Hydraulic head	Linear increasing	Higher hydraulic head = Higher suitability (1); lower values of hydraulic head = Lower suitability (risk of pumping difficulty)
Seawater intrusion index	Linear decreasing	Areas with low intrusion index = high suitability (1); areas with high intrusion index = low suitability (0)

This combined approach, weighted criteria from AHP and fuzzy membership reclassification of maps, ensures that both expert judgment and spatial variability are incorporated into the GIS-based suitability model. The methodology has been widely adopted in renewable energy and environmental planning studies^45,48^, demonstrating its robustness in handling both quantitative data and qualitative expert input.

### Data acquisition

The topographical, hydrogeological, economic, and other pertinent features of the study area shown in Fig. 4 form the basis of this investigation. Digital elevation model (DEM) data from the shuttle radar topography mission (SRTM) with a spatial resolution of 30 m were gathered from the USGS website for remote sensing applications. Elevation, slope, and land aspect criteria were calculated using this DEM data. Additionally, a solar radiation map was produced by combining the DEM data with the ArcGIS, the sources, formats, and resolutions of all datasets used in this study are summarized in Table 4.

**Table4:** Data collection source.

Data	Format	Source
Solar radiation	Raster	Derived using ArcGIS 10.8 solar radiation tool (based on DEM inputs)
Elevation	Raster	Shuttle radar topography mission (SRTM, 30 m) – USGS earth explorer
Slope	Raster	Calculated from DEM (ArcGIS 10.8 Software)
Aspect	Raster	Calculated from DEM (ArcGIS 10.8 Software)
Distance to roads	Vector	Extracted from national infrastructure datasets (ArcGIS 10.8)
Land cover (LULC)	Raster	Sentinel-2 imagery (Copernicus Open Access Hub, ESA)
Distance to shoreline	Raster	Derived from Sentinel-2 coastline extraction (Copernicus Open Access Hub, ESA)
Hydraulic head	Point	Field measurements collected during 2024 sampling campaign
Seawater fraction	Point	Hydrogeochemical/isotopic analysis from collected groundwater

### Sensitivity analysis methodology

To evaluate the repeatability and effectiveness of the suitability model derived from relative priority and normalized weights, a map removal sensitivity analysis was employed. This method systematically excludes one criterion at a time from the weighted overlay process and recalculates the suitability map. The resulting minimum and maximum suitability values are then compared against the baseline map.

### Sampling and analyses of water samples

A set of representative groundwater samples (51 of Quaternary, 47 of Upper Miocene and 58 of Lower Miocene) are collected during the first quarter of year 2024 from the northwestern coast of Egypt as illustrated in Fig. 1. Field measurements were done including physical parameters (electrical conductivity EC, pH, temperature, total dissolved solids TDS and dissolved oxygen DO) using portable probes. Chemical parameters comprising major cations (Ca, Mg, Na, and K) and anions (Cl, SO_4_, HCO_3_) concentrations were also collected in 1-L narrow neck pre–washed polyethylene bottles. Analysis of the water samples was carried out following the methods described in^49^. Total hardness (TH) as CaCO_3_ and Ca^2+^ were analyzed using standard EDTA. Mg^2+^ was calculated by taking the differential value between TH and Ca^2+^ concentrations. Na^+^ and K^+^ were measured using a flame photometer. Total alkalinity and CaCO_3_, CO_3_^2−^, and HCO_3_^–^ were estimated by titrating with HCl. Cl^−^ was determined by standard Hg (NO_3_)_2_ titration. SO_4_^2−^ and NO_3_^–^ was analyzed using UV/Visible spectrophotometer. All parameters are expressed in milligrams per liter and milliequivalents per liter. Data quality was assessed using the charge balance between the difference of cations and anions (expressed in meq/l) divided by their summation according to the following equation:

1 $${{\boldsymbol{\Sigma}}} \, \left( {{\mathbf{Cations}} - \, {\mathbf{Anions}}} \right)/{{\boldsymbol{\Sigma}}} \, \left( {{\mathbf{Cations}} + \, {\mathbf{Anions}}} \right) \, {\mathbf{X}} \, {\mathbf{100}}$$

With an acceptable range of ± 5^50^ that confirm the water quality assessment.

The stable isotopes measurements have been conducted for Oxygen -18, Deuterium according to the standard methods using Laser spectroscopy Picarro (Model L-2120i). Both ^18^O and D isotopic compositions are reported in permil (‰) deviation from isotopic standard reference material. The isotope ratios are generally represented in delta notation (δ).

## Results and discussion

### General hydrochemistry

The physical and chemical characteristics of groundwater samples collected from the Quaternary, Upper Miocene and Lower Miocene aquifers are summarised in Table 5, which presents minimum, maximum, mean and standard deviation values for each parameter alongside the World Health Organisation^51^ drinking water guidelines. Examination of these data reveals distinct hydrochemical signatures across the three aquifer systems.

**Table 5:** Statistical summary of measured physiochemical parameters for different aquifers in the study area.

		Quaternary aquifer			Upper Miocene aquifer			Lower Miocene aquifer		
Parameters		Min	Max	Mean	Min	Max	Mean	Min	Max	Mean
pH		6.3	7.4	6.75	6.4	7.8	6.84	6	7.7	6.61
EC	μs/cm	335	29,460	9261	777	22,380	7601	1150	62,850	12,451
TDS	mg/l	174	16,452	5377	505.89	12,884	4076	717.31	48,450	8113
Na	mg/l	9	4000	1192	85	2600	893	150	14,000	2158.17
K	mg/l	5	250	59.7	5	100	30.48	15	260	54.43
Ca	mg/l	31.2	832	232.5	25.64	458.6	249.6	22.64	1585	384.31
Mg	mg/l	17.69	1010.8	318.9	404.35	1011	722.78	27.51	2132	462.21
Cl	mg/l	29.4	9542	2524	146.81	5285	1821.51	245.1	26,792	3902.12
HCO_3_	mg/l	85.4	1769	364	73.2	800.93	287.93	85.4	647	297.32
SO_4_	mg/l	10	6945	1006	63.92	3600	842.43	0	4646	1149.24
TH		208	6225	1802	111	4769	1139	188	12,232	2363
*fsea* %		0	43.99	11.54	0.54	24.3	8.29	1	123.76	17.91
Calcite		-4.14	0.55	-0.47	-0.89	0.07	-0.41	-1.23	0.53	-0.47
Dolomite		-0.57	2.73	0.76	-0.82	1.77	0.54	-1.18	2.08	0.43
Anhydrite		-2.92	0.96	-1.37	-2.3	-0.84	-1.44	-2.57	-0.34	-1.29
Gypsum		-2.63	0.04	-1.14	-2.01	-0.55	-1.15	-2.28	-0.05	-1
Halite		-8.15	-3.29	-4.68	-6.45	-3.7	-4.71	-6.03	-2.35	-4.28
Barite		-1.42	1.16	0.27	-0.42	1.19	0.28	-0.66	0.92	0.17
Gibbsite		2.89	5.02	3.93	2.92	6.15	4.26	2.96	4.93	3.91
SAR		0.27	36.2	11.92	2.36	19.12	11.44	3.59	54.72	17.52
WQI		10.1	373.91	141.54	56.69	314.16	177.83	68.65	554.77	210.95

The pH values of groundwater from the Lower Miocene aquifer ranged from 6.0 to 7.7, with a mean of 6.61. In the Upper Miocene aquifer, pH varied between 6.4 and 7.8, averaging 6.84, while Quaternary aquifer samples exhibited pH values from 6.3 to 7.4 with a mean of 6.75. These ranges indicate slightly acidic to slightly alkaline conditions across all aquifers, potentially attributable to anthropogenic influences or natural biogeochemical processes. Electrical conductivity, a proxy for total dissolved ion concentration, demonstrated considerable variability both within and between aquifers. The Lower Miocene aquifer exhibited EC values from 1150 to 62850 μS/cm, with a mean of 12451 μS/cm. Upper Miocene aquifer samples ranged from 777 to 22380 μS/cm, averaging 7601 μS/cm, while Quaternary aquifer samples varied between 335 and 29460 μS/cm, with a mean of 9261 μS/cm. This wide range reflects substantial heterogeneity in groundwater mineralisation, likely resulting from variable degrees of aquifer interaction with saline sources. Total dissolved solids concentrations corroborated the EC patterns. Lower Miocene aquifer samples contained TDS between 717.31 and 48449 mg/l, mean 8112 mg/l. Upper Miocene aquifer values ranged from 505.58 to 12884 mg/l, mean 4076 mg/l, and Quaternary aquifer samples exhibited TDS from 174 to 16452 mg/l, mean 5377 mg/l. Comparison with WHO^51^ drinking water standards indicates that the majority of samples exceed permissible limits, with only 2, 7 and 8% of samples from the Lower Miocene, Upper Miocene and Quaternary aquifers respectively falling within acceptable ranges for potable use.

The major ion chemistry reveals sodium as the dominant cation, with mean concentrations of 2158.17 mg/l in the Lower Miocene, 893 mg/l in the Upper Miocene and 1192 mg/l in the Quaternary aquifer. Chloride similarly dominates the anion composition, with mean values of 3902.12 mg/l, 1821.51 mg/l and 2524 mg/l respectively. This sodium-chloride dominance suggests significant seawater influence or halite dissolution as primary salinity sources. Calcium and magnesium concentrations are also elevated, with mean calcium values of 384.31 249.6 and 232.5 mg/l across the three aquifers, and corresponding magnesium means of 462.21, 722.78 and 318.9 mg/l.

The seawater fraction calculations, based on chloride concentrations as a conservative tracer^52^, indicate substantial marine influence. Lower Miocene aquifer samples exhibited seawater fractions up to 123.76%, with a mean of 17.91%, suggesting some samples represent pure seawater or brines exceeding seawater concentration through evaporation. Upper Miocene aquifer samples reached 24.3% seawater fraction, mean 8.29%, while Quaternary aquifer samples attained 43.99%, mean 11.54%. These values confirm variable but widespread seawater intrusion affecting all three aquifer systems. Saturation indices calculated for relevant mineral phases provide insight into geochemical evolution. All aquifers are supersaturated with respect to dolomite, barite and gibbsite, indicating thermodynamic conditions favouring precipitation of these minerals. Conversely, undersaturation with respect to calcite, anhydrite, gypsum and halite suggests these phases remain undersaturated and continue to dissolve where present, contributing to ongoing mineralisation. Total hardness calculations following Hem (1985 classify the majority of samples as very hard. In the Quaternary aquifer, 7% of samples are hard and the remainder very hard; all Upper Miocene aquifer samples are very hard; and Lower Miocene aquifer samples, containing the highest seawater fractions, are uniformly very hard. These hardness characteristics have significant implications for both domestic and agricultural use.

The water quality index, computed from electrical conductivity, magnesium hazard, sodium adsorption ratio, sodium percentage, Kelly index and permeability index, provides an integrated assessment of suitability for various applications. Results indicate that 91% of Lower Miocene aquifer samples, 89% of Upper Miocene samples and 67% of Quaternary samples are categorised as unsuitable for drinking purposes, requiring appropriate treatment prior to use. A smaller proportion (5, 11 and 10% respectively) is classified as poor quality but potentially suitable for industrial and irrigation applications with appropriate management. Very poor quality, restricted to possible irrigation use only, characterises 3% of Lower Miocene and 8% of Quaternary aquifer samples.

#### Ionic relations and the origin of solutes

**Fig. 5:**
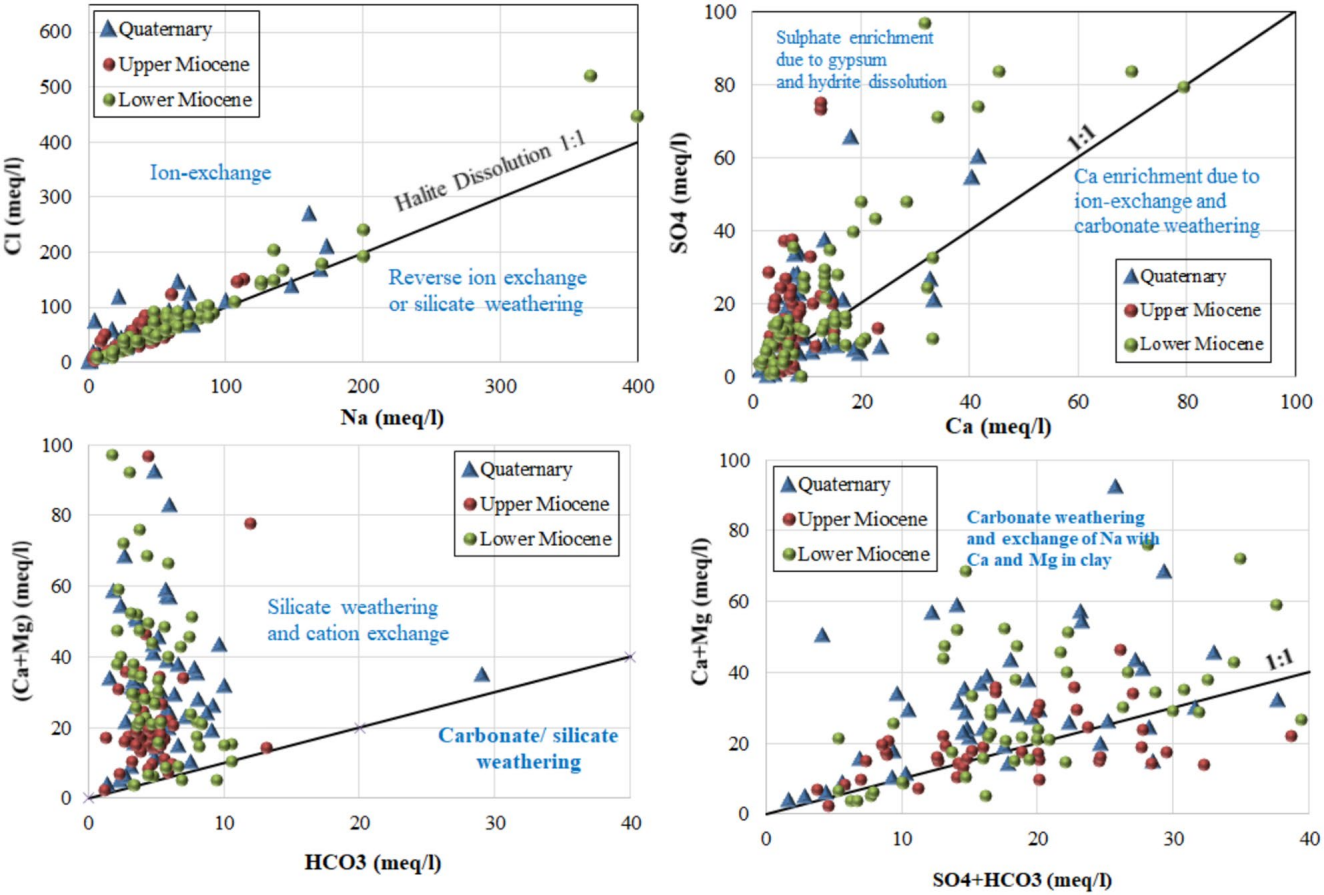
Ionic relationships for collected groundwater samples.

The stoichiometric relationships among dissolved ions provide diagnostic information regarding the sources of salinity and the geochemical processes operating within the aquifer systems. These relationships, examined through binary ionic plots, enable discrimination between seawater intrusion, mineral dissolution and ion exchange mechanisms. The relationship between sodium and chloride concentrations (Fig. 5a) exhibits a strong positive correlation (R^2^ = 0.773), indicating a common source or coupled behaviour. Samples plot both on and above the 1:1 halite dissolution line, with Na/Cl molar ratios ranging from 0.529 to 2.519 in the Lower Miocene aquifer, 0.245 to 1.285 in the Upper Miocene aquifer and 0.058 to 1.124 in the Quaternary aquifer. The elevated chloride concentrations relative to sodium in many samples suggest seawater influence, while sodium excess in others indicates additional sodium sources such as silicate weathering or cation exchange reactions. The relationship between sulphate and calcium (Fig. 5b) informs assessment of gypsum and anhydrite dissolution. Where sulphate exceeds calcium on an equivalent basis, the implication is that calcium has been removed from solution through precipitation of calcite or participation in ion exchange reactions. Examination of this relationship across the dataset reveals variable stoichiometry, with some samples exhibiting sulphate excess consistent with calcium removal processes. The (calcium + magnesium) versus bicarbonate relationship (Fig. 5c) demonstrates clustering of most samples near the 1:1 line, suggesting that dissolution of atmospheric carbon dioxide in fresh water contributes to the acquisition of these ions. However, in many samples, particularly those from deeper wells with elevated salinity, calcium and magnesium exceed bicarbonate concentrations, indicating additional sources of these divalent cations beyond carbonate dissolution.

#### Processes controlling groundwater salinization

**Fig. 6:**
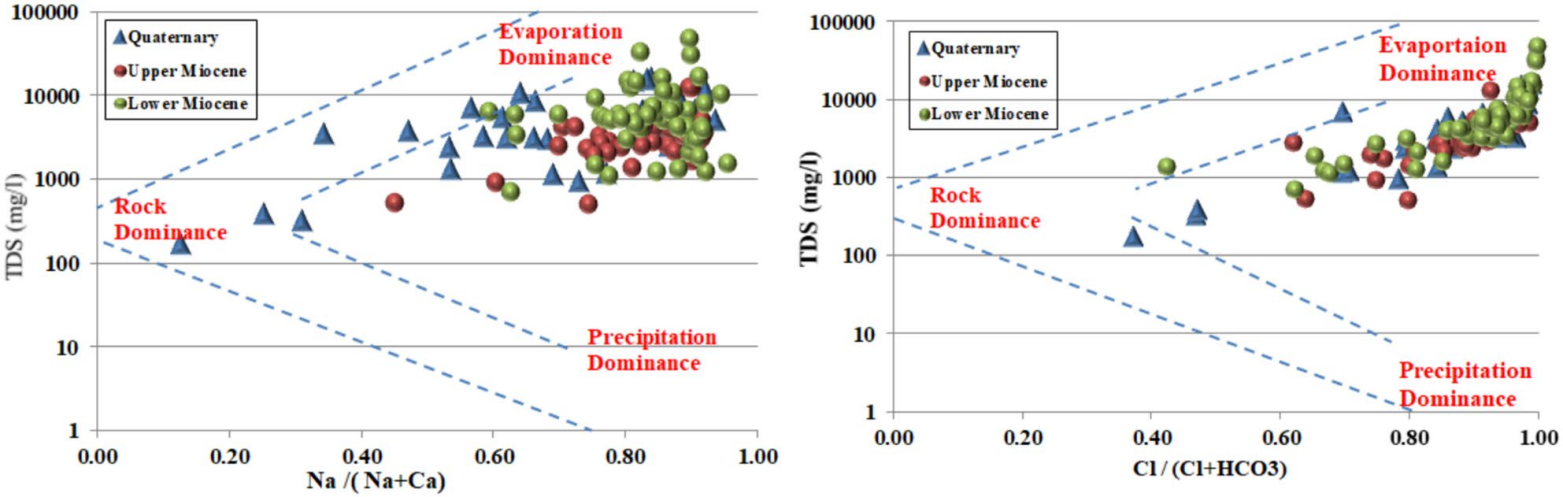
Gibbs plot for collected water samples.

The Gibbs diagram^53^ provides a conceptual framework for distinguishing the dominant natural mechanisms governing groundwater chemistry: atmospheric precipitation, rock weathering and evaporation-crystallisation. Plotting the collected groundwater samples on Gibbs diagrams (Fig. 6) reveals that evaporation and rock-water interaction constitute the primary processes controlling solute acquisition across the study area. Samples from all three aquifers plot predominantly within the evaporation dominance field and the transition zone toward rock weathering dominance. This distribution indicates that evaporative concentration, whether occurring prior to recharge or within the aquifer system itself, plays a substantial role in elevating salinities. The absence of samples plotting within the precipitation dominance field confirms that atmospheric inputs are not the primary control on groundwater chemistry in this region.

**Fig. 7:**
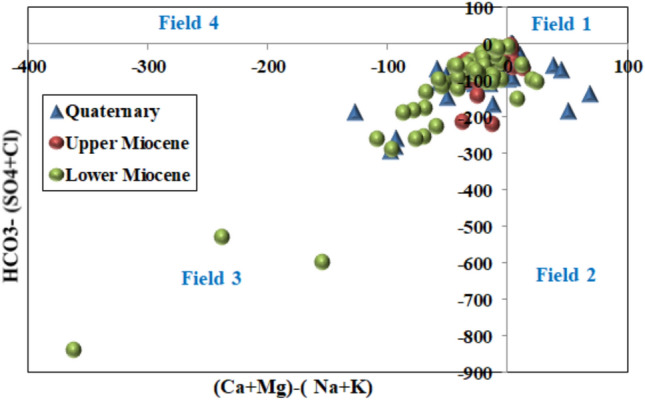
Chadha diagram for collected water samples.

The Chadha diagram^54^, a modified version of the Piper diagram, enables classification of groundwaters according to their hydrochemical facies. This rectangular diagram is divided into eight sub-fields representing distinct water types based on the differences between alkaline earths and alkali metals on the abscissa and between weak acidic anions and strong acidic anions on the ordinate. As illustrated in (Fig. 7), the majority of groundwater samples from the study area fall within Field 3, characterised by alkali metals exceeding alkaline earths, with sodium dominance among cations and chloride dominance among anions. This hydrochemical facies is typical of waters influenced by seawater intrusion or halite dissolution, consistent with the elevated sodium and chloride concentrations documented in Table 2. A secondary group of samples occupies Field 2, where calcium and magnesium are the dominant cations, indicating zones where carbonate dissolution exerts greater influence on water chemistry.

#### Sources of salinity and its associated process on water chemistry

Cation exchange reactions are intimately associated with salinisation processes in coastal aquifers, as documented extensively in the literature^55,56^ These reactions modify the relative proportions of cations in solution as fresh water and salt water interact, with enrichment or depletion of specific cations providing diagnostic indicators of exchange direction and intensity. The seawater fraction calculations presented in Section "General hydrochemistry", based on chloride as a conservative tracer^52,57^, quantify the extent of marine influence in each sample. The substantial seawater fractions observed, particularly in the Lower Miocene aquifer where values reach 123.76%, confirm that seawater intrusion constitutes a primary salinisation mechanism. Values exceeding 100% indicate samples that have undergone evaporative concentration beyond simple seawater mixing, consistent with the evaporation dominance indicated by Gibbs diagrams. Saturation indices computed for relevant mineral phases provide additional constraints on geochemical evolution. The observed supersaturation with respect to dolomite, barite and gibbsite, coupled with undersaturation for calcite, anhydrite, gypsum and halite, indicates that precipitation of the former minerals may occur while the latter phases remain capable of dissolution where present. This combination of saturated and undersaturated phases reflects the complex, multi-source evolution of groundwater salinity in these aquifer systems. The water quality categorisation presented in Table 6 synthesises the hydrochemical data into an integrated assessment of suitability for various applications. The predominance of unsuitable classifications across all aquifers (91% Lower Miocene, 89% Upper Miocene, 67% Quaternary) reflects the combined effects of seawater intrusion, evaporative concentration and mineral dissolution processes documented throughout this analysis. The small proportions of poor and very poor-quality water, potentially suitable for irrigation with appropriate management, are restricted to samples exhibiting lesser degrees of saline influence.

**Table 6:** Water quality index categorization for groundwater samples of the study area.

Aquifer	Total no. of samples	Unsuitable	%	Poor	%	Very poor	%
Quaternary	51	34	67	5	10	4	8
Upper Miocene	47	42	89	5	11		
Lower Miocene	58	53	91	3	5	2	3

### Aquifer-specific isotopic characteristics and implications for site suitability

Stable environmental isotopes (δ^1^⁸O and δD) serve as intrinsic tracers within the hydrological cycle, with their relative abundances modified by physical processes including evaporation, condensation and mixing. These variations provide diagnostic information regarding groundwater origin, recharge mechanisms and interactions between different water bodies. In the context of site suitability assessment for green hydrogen production, the isotopic composition of groundwater informs the availability and sustainability of freshwater resources required for electrolysis, as well as the extent of seawater intrusion that may compromise water quality. Stable environmental isotopes (δ^1^⁸O and δD) are intrinsic constituents of water molecules, with their relative abundances altered by physical and mass-dependent processes during the hydrological cycle. These variations reflect the origin, movement, and interactions of water within hydrogeological systems. The isotopic composition of groundwater is influenced by meteorological conditions (e.g., temperature, humidity), source-specific signatures (rainfall, surface water, seawater), and secondary processes (evaporation, mixing). In the study area, potential recharge sources exhibit distinct isotopic fingerprints (Table 7):

**Table 7:** Isotopic signatures of potential recharge sources in the study area.

Source	δ^1^⁸O (‰)	δD (‰)	d-excess	Reference
Paleowater from the Nubian Sandstone Aquifer	−10	−80	0	^58^
Mediterranean seawater	1.76	11.92	−2.16	^59^
Local precipitation	Marsa Matruh rainfall: −3.24 Alexandria rainfall: −4.52			**GNIP/IAEA data**

The isotopic composition of groundwater exhibits distinct signatures across the three aquifer systems, reflecting differences in recharge history, hydraulic connectivity and dominant geochemical processes. These variations carry direct implications for the availability and sustainability of water resources for green hydrogen production.

#### Quaternary aquifer

**Fig. 8:**
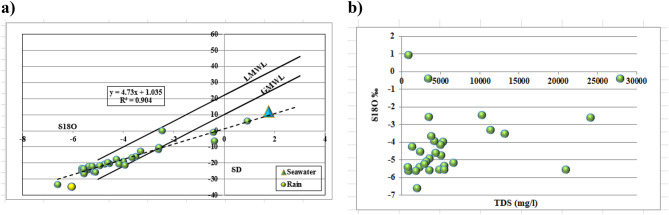
Binary relationship for quaternary aquifer between; (**a**) δ^1^⁸O and δD and (**b**) δ^18^O (‰) and TDS (mg/l).

Groundwater from the Quaternary aquifer exhibits mean δ^1^⁸O of − 4.16‰ and mean δD of − 18.65‰ (Table 8), values substantially depleted relative to modern Mediterranean precipitation (δ^1^⁸O: − 3.24‰; δD: − 11.25‰). This depletion indicates that a significant component of recharge occurred under cooler and/or wetter climatic conditions than those prevailing today. The observed range (δ^1^⁸O: − 6.60 to 0.93‰; δD: − 33.50 to 5.84‰) reflects mixing between paleowater and recent recharge, with the most enriched samples (δ^1^⁸O approaching 0.93‰) indicating evaporative modification in shallow zones or mixing with saline sources. The δ^1^⁸O-δD relationship (δD = 4.731 δ^1^⁸O + 1.0357; R^2^ = 0.9048) yields a slope considerably lower than the Global Meteoric Water Line (GMWL: δD = 8 δ^1^⁸O + 10;^60^), demonstrating that evaporation has modified the isotopic composition prior to or during recharge. The strong linear correlation (R^2^ = 0.9048) indicates a consistent evaporative regime across the aquifer, with samples representing varying degrees of enrichment along a common evaporation trajectory. The positive correlation between δ^1^⁸O and TDS (Fig. 8b) is consistent with evaporative concentration, wherein water loss through evaporation simultaneously enriches residual water in heavy isotopes and increases dissolved solute concentrations. Samples approaching the seawater end-member (δ^1^⁸O =  + 1.76‰; TDS = 35,000 mg/l) delineate zones where marine intrusion contributes to groundwater salinisation.

**Table 8:** Statistical analyses of (δ^18^O and δD) isotopic values of Marsa Matruh governorate samples.

Aquifer	Item	St Dev	Kurtosis	Skewness	Minimum	Maximum	Mean
Quaternary	δ^18^O (‰)	2.88	-0.34	1.04	-6.60	0.93	-4.16
Upper Miocene	δD (‰)	16.60	0.25	1.25	-33.50	5.84	-18.65
Marmarica (Lower Miocene (	δ^18^O (‰)	3.48	-0.03	0.88	-10.35	-0.48	-4.73
	δD (‰)	23.40	0.99	0.15	-80.29	-1.98	-24

#### Miocene aquifers

The Miocene aquifers display more depleted isotopic signatures than the Quaternary aquifer, with mean δ^1^⁸O of − 4.73‰ and mean δD of − 24.00‰ for both Upper and Lower units (Table 8). The most depleted samples (δ^1^⁸O: − 10.35‰; δD: − 80.29‰) approach the composition of Nubian Sandstone paleowater (δ^1^⁸O: − 10.0‰; δD: − 80.0‰), suggesting that deep circulation from the regional aquifer system contributes to Miocene groundwater in some areas. The wide range in δD values (standard deviation 23.40‰) relative to δ^1^⁸O (3.48‰) reflects variable mixing between isotopically distinct end-members.

**Fig. 9:**
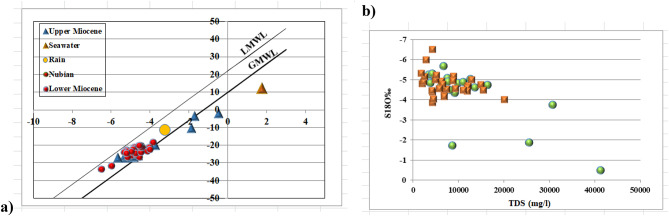
Binary relationship for Miocene aquifer (lower and upper) between; (**a**) δ^1^⁸O and δD and (**b**) δ^18^O (‰) and TDS (mg/l).

The δ^1^⁸O-δD relationships for Miocene samples, Fig. 9a) define two distinct regression lines: Upper Miocene (δD = 5.0387 δ^1^⁸O + 1.1504; R^2^ = 0.8507) and Lower Miocene (δD = 4.5369 δ^1^⁸O − 2.0142; R^2^ = 0.8198). Both slopes are lower than the GMWL, indicating evaporative influence, though the Upper Miocene samples exhibit a slightly higher slope, suggesting less evaporative modification or greater contribution from depleted paleowater. The Lower Miocene regression line exhibits a negative intercept (− 2.0142), consistent with mixing between evaporated modern water and a depleted paleowater component. The elevated R^2^ values demonstrate that mixing between end-members, rather than random variation, controls the observed isotopic distribution. The relationship between δ^1^⁸O and TDS for Miocene samples (Fig. 9b) differs markedly from the Quaternary aquifer. Despite a wide TDS range (approximately 2000 to > 40,000 mg/l), δ^1^⁸O values remain relatively constrained between − 6‰ and − 2‰. This pattern indicates that salinity acquisition in the Miocene aquifers occurs primarily through water–rock interactions—including dissolution of evaporite minerals, cation exchange and mixing with connate brines—rather than evaporative concentration. The absence of a strong δ^1^⁸O-TDS correlation suggests that saline samples have acquired their dissolved solids through subsurface processes that do not fractionate oxygen isotopes.

### Synthesis and site suitability implications

The isotopic evidence yields three principal findings relevant to site suitability assessment for green hydrogen production:

First, the Quaternary aquifer receives measurable modern recharge, as evidenced by isotopic values extending toward the local meteoric water line and the positive δ⁸O-TDS correlation indicative of ongoing evaporative processes. However, elevated salinities in coastal zones and isotopic evidence for seawater mixing indicate that groundwater quality is compromised near the Mediterranean shoreline. Sites underlain by the Quaternary aquifer at sufficient distance from the coast may access water suitable for electrolysis following appropriate treatment, though the evaporative signature in shallow groundwater implies vulnerability to seasonal and climatic variations in recharge quality.

Second, the Miocene aquifers contain predominantly paleowater with limited modern recharge, as indicated by depleted isotopic signatures and the absence of an evaporation-TDS relationship. The most depleted samples approach the composition of Nubian paleowater, suggesting that deep, fossil groundwater contributes to these aquifers. While water quality is highly variable, the deep, confined nature of the Lower Miocene aquifer may provide a more stable and protected resource, albeit one that is effectively non-renewable on human timescales. Any abstraction for hydrogen production would require careful assessment of sustainable yield and consideration of the non-renewable nature of the resource.

Third, isotopic evidence for seawater intrusion in coastal portions of both aquifer systems delineates zones where groundwater is unsuitable for electrolysis without substantial desalination. The mixing relationships quantified through δ^1^⁸O-δD regression lines provide a basis for mapping the extent of marine influence and identifying areas where fresh groundwater remains protected. Samples exhibiting δ^1^⁸O values above −2‰ and elevated TDS are clearly influenced by seawater and should be avoided for hydrogen production without desalination infrastructure.

These hydrochemical and isotopic constraints, when integrated with the economic and environmental criteria discussed previously, inform the identification of sites where adequate water resources of suitable quality can be secured for long-term hydrogen production operations. The evidence suggests that optimal sites will be located inland, away from the coastal seawater intrusion zone, and underlain by aquifers with sufficient freshwater reserves to support sustained abstraction.

## Multicriteria site suitability for a solar-powered green hydrogen production

**Fig. 10:**
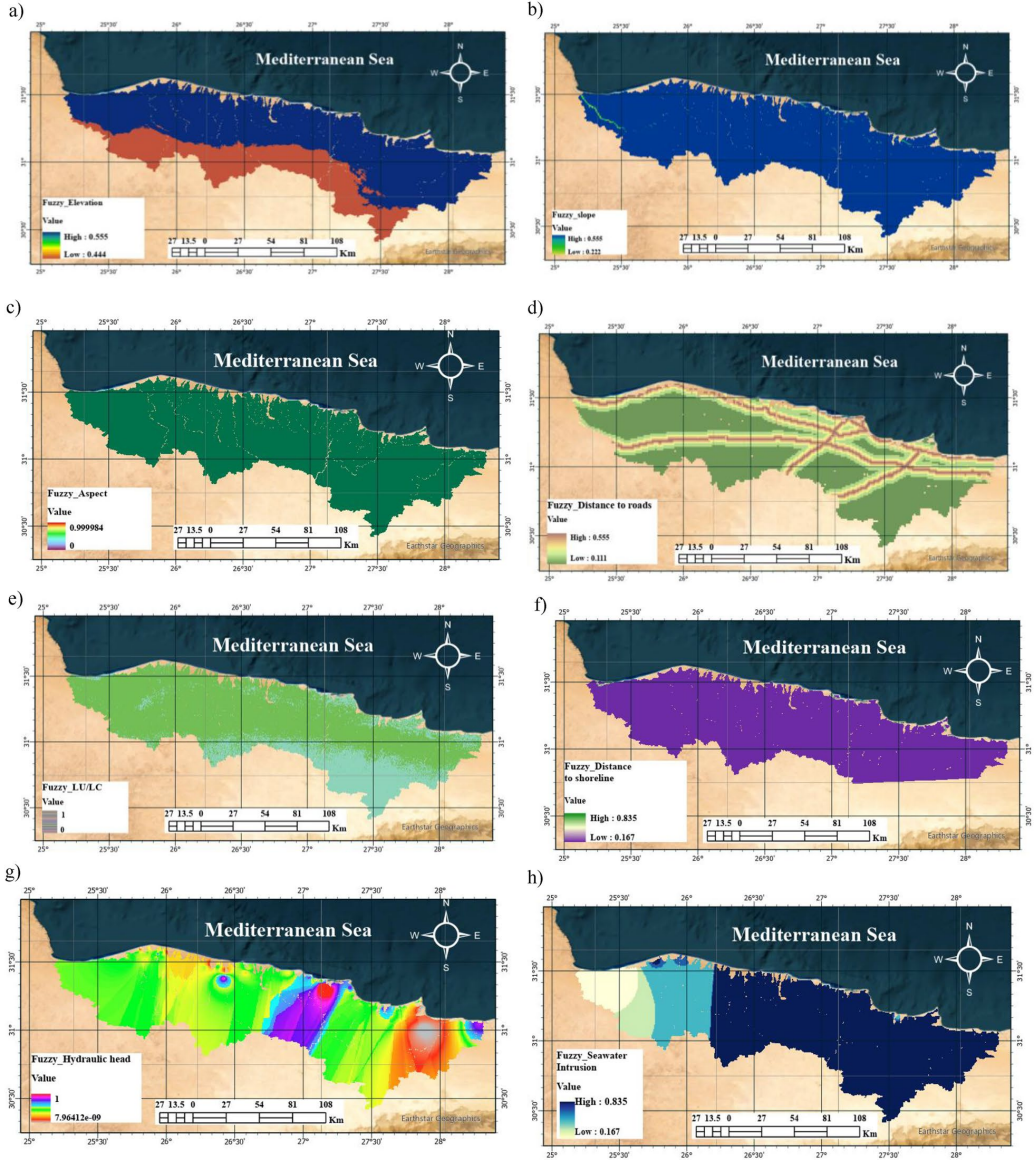
Fuzzy based criteria for Marsa Matruh site (the maps were created using QGIS (QGIS Development Team, 2025, Version: 3.34.6-Prizern. Available at: https://qgis.org).

Fuzzy-AHP as a multi-criteria decision-making approach enables decision-makers to assess the relative importance of various criteria and alternatives in scenarios involving uncertainty or imprecision the relative fuzzy membership for each thematic map was acquired using Arc GIS as indicated in (Fig. 10a–h). The fuzzy membership maps, which standardize each criterion to a common scale (0 = least suitable, 1 = most suitable), reveal distinct spatial patterns across the study area between the coast and the southern hinterlands.

- **Elevation** (Fig. 10a): The fuzzy elevation surface exhibits a clear north–south gradient. Suitability increases from the southern inland areas (fuzzy value = 0.444) towards the Mediterranean coast (0.555). This pattern reflects the marginal reduction in atmospheric mass at higher elevations, which slightly diminishes solar irradiance and, consequently, the potential yield of photovoltaic infrastructure.
- Slope (Fig. 10b): Terrain slope presents a more restrictive constraint. The fuzzy slope values range from 0.222 in the steeper, dissected terrain to 0.555 in the flatter coastal plains and depressions. This distribution is critical because low-slope areas minimize the need for extensive grading and earthworks, reducing both construction costs and long-term erosion risks for large-scale installations.
- Aspect (Fig. 10c): The map highlights how terrain orientation (aspect) influences site suitability for solar-powered green hydrogen production. South-facing slopes generally receive greater solar radiation, thus scoring higher in the fuzzy classification. Conversely, north-facing slopes are less favorable due to reduced solar exposure.
- Distance from roads (Fig. 10d): Accessibility, quantified as fuzzy distance to the road network, shows a steep gradient in suitability (0.111 to 0.555). Proximity to existing transport corridors is a strong determinant of economic feasibility, as it directly offsets the capital expenditure associated with constructing new access roads for heavy equipment and hydrogen transport.
- Land use/land cover (Fig. 10e): The land use map delineates zones of environmental constraint. Built-up areas, irrigated agriculture, and potential ecological zones were deprioritized. The analysis strongly favours barren lands and desert surfaces, which align with higher fuzzy suitability values, thereby minimizing land-use conflict and adhering to environmental safeguarding principles.
- Distance to shoreline (Fig. 10f): Proximity to the Mediterranean Sea is essential for seawater intake. The fuzzy distance to shoreline values increases sharply from 0.167 at inland locations to 0.835 along the immediate coast. While this underscores the logistical advantage of coastal sites, it is imperative to cross-reference these locations with flood hazard assessments to ensure infrastructure resilience.
- Hydraulic head (Fig. 10g): The hydraulic head surface, ranging from −21.3 to + 34.3 m, provides insight into coastal groundwater dynamics. The negative values observed in specific localities are indicative of a reversed hydraulic gradient, a condition that facilitates the landward encroachment of saline water and signals potential constraints on groundwater availability.
- Seawater intrusion index (Fig. 10h): This index synthesizes the vulnerability of coastal aquifers, with fuzzy values spanning from 0.167 (low risk) to 0.835 (high risk). Zones characterized by high intrusion risk constitute a long-term environmental constraint, as they limit the use of fresh groundwater for auxiliary plant operations and pose a risk of subsurface infrastructure corrosion.

**Fig. 11:**
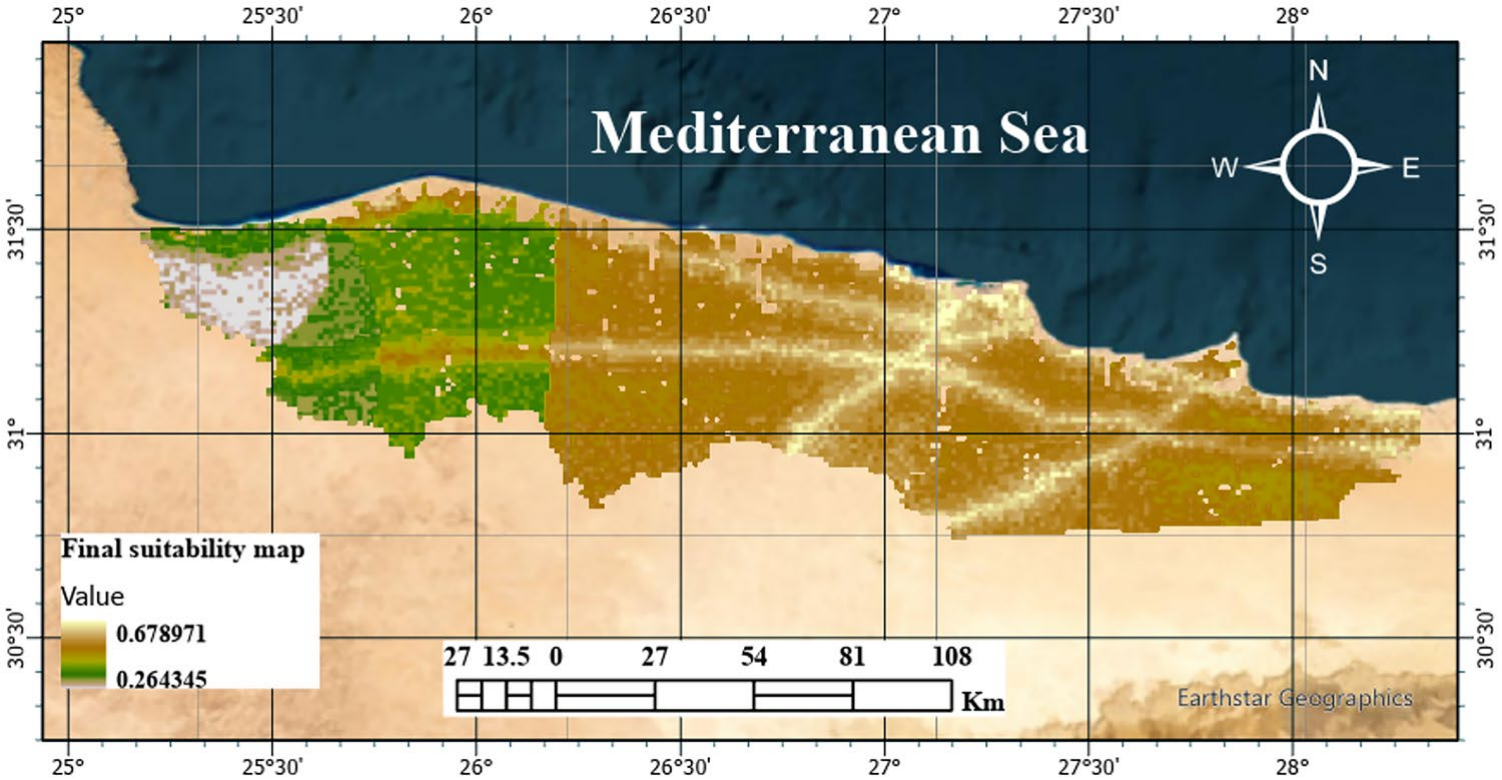
Final site suitability map for green-hydrogen production (the map was created using QGIS (QGIS Development Team, 2025, Version: 3.34.6-Prizern. Available at: https://qgis.org).

The final suitability map in Fig. 11; represents the integrated output of the multicriteria evaluation for solar-powered green hydrogen production along the coast. Suitability values range between 0.264 and 0.679, with lower values indicating marginal suitability and higher values denoting favourable conditions for infrastructure development. The spatial distribution shows that coastal plains and low-lying areas near the Mediterranean shoreline consistently achieve higher suitability scores. These zones benefit from flat terrain, accessibility, and proximity to seawater resources, which are essential for hydrogen production processes. In contrast, inland regions with higher elevation, steeper slopes, or land cover constraints exhibit lower suitability values. The clustering of high-suitability areas along the coast underscores the importance of integrating multiple spatial criteria, slope, aspect, elevation, land use/land cover, proximity to roads, shoreline distance, hydraulic head, and seawater intrusion index, through the fuzzy-AHP framework. This integration allows for a balanced assessment that accounts for both technical feasibility and environmental considerations. Based on the above discussion, Fig. 11 provides a clear decision-support tool, highlighting priority zones for hydrogen plant development. It demonstrates the most promising sites, in addition to the environmentally sensitive or topographically constrained areas that should be avoided.

### Sensitivity analysis

Each parameter, elevation, slope, aspect, distance to roads, distance to shoreline, seawater intrusion index, hydraulic head, and land use/land cover, was removed individually. The recalculated maps provided new ranges of suitability values, allowing assessment of the structural dependence of the model on each parameter. Criteria whose removal caused substantial reductions in maximum suitability values as indicated in Table 9 (e.g., distance to shoreline and seawater intrusion index) were identified as dominant drivers. Conversely, criteria whose removal resulted in minimal changes or slightly higher maximum values (e.g., slope, aspect, elevation) were considered as constraints that limit suitability rather than drivers. Parameters with intermediate effects (e.g., hydraulic head, distance to roads, LULC) were classified as moderate contributors.

**Table 9:** Sensitivity analysis results (baseline vs. map removal).

Parameter Removed	Baseline Min	Baseline Max	Removal Min	Removal Max	ΔMin (%)	ΔMax (%)	Influence Interpretation
Elevation	0.264	0.679	0.170	0.583	−35.6	−14.1	Constraint, reduces suitability
Slope	0.264	0.679	0.241	0.650	−8.7	−4.3	Constraint, minor effect
Aspect	0.264	0.679	0.258	0.650	−2.3	−4.3	Constraint, minor effect
Distance to roads	0.264	0.679	0.199	0.612	−24.6	−9.9	Moderate contributor
Distance to shore	0.264	0.679	0.197	0.536	−25.4	−21.0	Dominant driver
Seawater fraction	0.264	0.679	0.214	0.495	−18.9	−27.1	Dominant driver
Hydraulic head	0.264	0.679	0.203	0.602	−23.1	−11.3	Moderate contributor
Land use / land cover	0.264	0.679	0.204	0.602	−22.7	−11.3	Moderate contributor

The analysis ensures that the final suitability map is not overly dependent on subjective weight assignments and remains structurally robust for decision-support in environmental planning.

## Comparison with similar studies

The methodological framework employed in this study is consistent with a growing body of literature that integrates GIS with MCDM techniques for renewable energy infrastructure planning. The combination of AHP for criteria weighting and spatial overlay analysis in ArcGIS follows approaches documented across diverse geographical settings, including the Konya region in Türkiye^61^, Eastern Morocco^62^, Amrani et al., 2023), Pakistan^63^, and Vietnam^64^. The recurrence of this integrated approach across multiple contexts underscores its suitability for addressing the complex trade-offs inherent in hydrogen infrastructure site selection. The criteria selected in this study, technical (solar irradiance, slope), economic (road proximity), environmental (land use, seawater intrusion), and infrastructural factors, are broadly comparable to those reported in previous investigations. Studies conducted in the Southern Marmara region of Türkiye^65^. However, the explicit incorporation of hydrogeological constraints, specifically hydraulic head and seawater intrusion index, represents a context-specific adaptation reflecting the coastal aquifer dynamics along Egypt’s northwest coast. This dimension is less commonly addressed in arid-region studies, which typically emphasize surface water availability or distance to desalination facilities rather than subsurface saline intrusion risks^66^. Recent studies have extended the conventional AHP-GIS framework through scenario analysis examining decentralized versus centralized production configurations^62,67^ and through systematic sensitivity testing of weight assignments^16,68^. While the present study establishes baseline suitability classifications, future iterations could incorporate these methodological extensions to examine how alternative weighting schemes or production scales might influence the spatial distribution of suitable zones. Comparison with findings from Pakistan^63^ and Türkiye^69^ reveals a consistent pattern: coastal zones with gentle topography and existing infrastructure access consistently emerge as highly suitable, whereas inland areas with high solar irradiance but limited water availability or difficult terrain are generally deprioritized. This recurring trade-off underscores the importance of localized criteria weighting and the necessity of adapting generic frameworks to regional environmental and infrastructural realities^70^.

## Conclusion

This study demonstrates that integrating geographic information systems (GIS) with multi-criteria decision making (MCDM) provides a robust framework for identifying suitable sites for solar-based green hydrogen production along Egypt’s northwest coast. By incorporating technological, economic, and environmental criteria, the approach ensures that site selection is both systematic and transparent, facilitating stakeholder engagement throughout the decision-making process. The application of the analytic hierarchy process (AHP) enabled the quantification of criterion weights, revealing the relative importance of factors such as solar irradiation, slope, proximity to roads, and distance from the shoreline. Overlaying these weighted criteria in ArcGIS produced a suitability map classifying the study area into five distinct zones: very low, low, moderate, high, and very high suitability. The results indicate that the most favourable sites are concentrated in low-elevation coastal zones with gentle slopes, existing road access, and minimal environmental constraints, while areas with steep terrain, high seawater intrusion risk, or sensitive land cover were deprioritized. Several limitations should be acknowledged. First, the analysis relied on publicly available datasets (e.g., SRTM DEM at 30 m resolution), which may not capture fine-scale topographic or land-use variations. Second, the AHP method, while widely used, involves subjective judgments in pairwise comparisons that could influence outcomes. Third, dynamic factors such as future infrastructure development should be considered. Future research should focus on integrating higher-resolution remote sensing data to refine suitability assessments at the local scale. Incorporating temporal dimensions, such as variability in solar irradiance or projected sea-level rise, would enhance the site selection process. Additionally, combining this framework with life cycle assessment (LCA) and techno-economic modelling could provide a more holistic evaluation of project viability. Finally, engaging local stakeholders through participatory GIS approaches would ensure that social and cultural factors are adequately represented in future iterations of this work.

## Data Availability

All data generated or analyzed during this study are included in this manuscript. All authors have read, understood, and have complied as applicable with the statement on “Ethical responsibilities of Authors” as found in the Instructions for Authors and are aware that with minor exceptions, no changes can be made to authorship once the paper is submitted.
